# Supplementary material for: How do hospital goals resonate with leaders, clinicians, managers, and patient and family partners? A critical discourse analysis of institutional logics
Source: PLoS One. 2026 Mar 18;21(3):e0344582. doi: 10.1371/journal.pone.0344582 (PMC12998869; doi:10.1371/journal.pone.0344582)
Supplement: S1 File — (DOCX) [file pone.0344582.s001.docx]

**Supplementary File**

How do Hospital Goals Resonate with Leaders, Clinicians, Managers, and Patient and Family Partners? A Critical Discourse Analysis of Institutional Logics

[Interview Guide 2](#_Toc212112086)

[Participant Characteristics 6](#_Toc212112087)

[Healthcare Logics in Organizational Documents 7](#_Toc212112088)

[Healthcare Logics Across Participant Groups 8](#_Toc212112089)

# Interview Guide

Thank you for taking the time to talk to me today. This conversation may take anywhere between 30-60 minutes but can be longer or shorter depending on your preference or how much time you have.

This study focuses on patient and family engagement in hospitals. We define patient and family engagement as ways in which patients and other stakeholders draw on patient experience to inform health care activities such as how health services are designed and delivered. Many hospitals have goals for patient engagement (PE) activities and being more person-centered. It is not always clear if these goals translate into day-to-day activities. These goals are articulated by hospitals in their mission, vision, and value statements. Specifically, I am interested in understanding the connection between hospital mission, vision, and value statements as it relates to PE and what happens in the day-to-day activities at the hospital.

Do you give your consent to audio-record this conversation?

[start recording]

*Confidentiality -* Before we begin our discussion, I want to spend a few moments going over a few key points about the interview today:

- Your participation is voluntary. You can leave or stop participating in this interview at any moment you choose with no repercussions on yourself.
- You can skip any questions.
- The information which we collect from these interviews will unlikely be connected to you. We will likely use quotes from your interviews, and when we do so we will change your name. For example, we will use “patient partner” to refer to your quotes.
- If we decide we want to use a quote and attribute it to you, we will contact you ahead of time to see if that was all right with you.
- I will strive to protect the confidentiality of our discussion or your written responses.

*Use of Tape Recorder –* this interview will be recorded to support transcription and analysis with your permission.

- All audio files and transcripts will be kept in a secure folder accessible to only the primary investigators.
- I will also be taking notes throughout the conversation.
- Only the research team will have access to transcripts from this conversation.
- The tapes and transcripts will only be used for this project.

When using abbreviations or acronyms, we ask that you say the full name at least once to aid transcription.

When talking about patient and family engagement, note that I will also be interested in activities and approaches related to patient experience, patient-centered care, and related concepts.

If at any point you feel tired or fatigued, please let us know and we can take a short break. I may also ask periodically if you would like to take a short break.

Do you have any questions before we begin?

Do you give your consent to participate in this interview?

1. Tell me a bit about yourself including your role at the hospital.

Probes:

- - How long have you been here?
  - Have you served any other roles at the hospital?
  - Have you served roles at other hospitals?

1. Can you walk me through patient and family engagement at your hospital?
   - From your perspective, is patient and family engagement important to the hospital?
   - Can you share an example of how the hospital expresses the importance of patient and family engagement? In what ways is patient and family engagement encouraged at the hospital?

Optional Prompts:

- - - Tell me a bit more about that.
    - Can you give an example showing how important patient and family engagement is to the hospital?
  - Can you talk to me about how patient and family engagement has been encouraged at the hospital?

Optional Prompts:

- - - What activities, approaches, and strategies have been implemented to encourage patient and family engagement?
    - Can you give me an example?
  - Can you share with me any formal goals in your hospital that relate to patient and family engagement?

Optional Prompts:

- - - What are your hospital’s goals in relation to patient and family engagement?
      - Were staff and partners involved in the development of your hospital’s patient and family engagement goals? How were they involved?
      - Can you provide an example of how they were involved?
    - Are you part of any hospital committees or planning groups where the goals of the hospital were/are discussed?
      - What were the objectives of that committee/group?
      - Do any of these goals relate to patient and family engagement? Tell me more…
- How did you feel about the removal of the mental health PFAC?
  - - Do you know what prompted this change?
    - In what ways has patient and family engagement in mental health activities changed as a result of the removal of the mental health PFAC?
      - Can you give me an example of what that engagement is like now?
      - Is this the same/different as before?

[FIRST] ASK THEM ABOUT REALIZATION FACTORS IN GENERAL TO UNDERSTAND WHAT THEY THINK IS IMPORTANT FOR REALIZING ORGANIZATIONAL GOALS.

- - [communication] I’d like to ask you a bit now about how your organization communicates about patient and family engagement. Can you walk me through how communication about patient and family engagement goals happens at the hospital?
    - [ask about the alignment of goals across documents in different departments/units]
    - Have you seen a misalignment between goals and practices? Tell me more…
    - How do you communicate different types of goals? For example, business goals, community goals, quality of care goals, and patient experience goals.

Optional Prompts:

- - - Do you think it is important to communicate organizational goals related to patient and family engagement to staff and partners? Why?
    - Have any frameworks been developed to guide patient and family engagement?
      - Ask about the patient bill of rights and responsibilities
      - Why were they developed?
      - What were their outcomes?
    - Has the executive team at the hospital communicated patient and family engagement goals and values to you?

If yes: how did it happen? Can you provide an example?

- - - - - How do patient and family partners interact or communicate with hospital leadership (i.e., executives, managers, clinical managers)?
        - What strategies have they used to communicate goals to hospital staff, patients, and family partners?
        - In your view, have they worked? Why or why not?
        - Are there any strategies that have not been used but you believe would effectively communicate patient and family engagement goals and values?
        - Do you believe that the diversity of patients and families was considered when communicating patient and family engagement goals and values? Tell me more…

If no: do you think communicating patient engagement goals is important? Tell me more…

- - [personal factors] Tools, resources, and supports (e.g., space, funding, mentorship)can play an important role in promoting patient engagement. Can you talk to me about what resources and supports are available to you for patient and family engagement?
    - Is the hospital aware of any barriers you face (transportation, health literacy, illness, time, and caregiving responsibilities)? Tell me more…
      - Does the hospital provide needed support so that you can participate in the way you want to?
      - What would make it easier for you to participate in patient and family engagement activities?

Optional Prompts:

- - - Can you give an example?
    - [alignment] Can you walk me through how your hospital has set up or changed organizational structures and health care activities/processes to support patient and family engagement goals and values? Tell me more about that.
      - [alignment] In your view, is the hospital doing specific things to promote and show examples of valuing patient and family engagement? Can you give an example?
      - [alignment] Have there been any changes to the types of health care activities that patients and families can engage in at the hospital? Tell me more…
      - Have there been any new celebrations or activities that you can engage in?
      - Has there been staff dedicated to patient and family engagement? Tell me more…
      - In your view, how have these changes impacted patient and family engagement?
  - [motivation and empowerment] Finally, I want to know more about your thoughts on how staff and partners are motivated and empowered towards patient and family engagement at the hospital.

Optional Prompts:

- - - Do you think it is important to motivate and empower staff and partners towards patient and family engagement? Why?
    - How has the leadership team motivated or empowered staff and partners to participate or lead patient and family engagement activities (leaders may include executives, managers, and clinical leaders)?
      - Can you give a specific example?
    - What has the leadership or executive team done to exemplify your hospital’s goals and values for patient engagement?
      - Can you give a specific example?

**Diversity and Inclusion**

- Would you say that equity, diversity, and inclusivity are important for patient and family engagement at the hospital? Why or why not? (Emphasize: diversity, such as ethnicity and socioeconomic status)
- In your view, do the patient and family partners at your hospital reflect the diversity of the community the hospital serves? Tell me more.

Thank you for taking the time to participate in this interview. Before we close off, do you have any questions or anything else you would like to express?

[stop recording]

Is there anyone whom we should interview? Can you connect me with them?

# Participant Characteristics

|  | **Role** | **Years at Hospital** | **Age (years)** | **Gender** | **Highest Education** | **Ethnicity/Race** |
| --- | --- | --- | --- | --- | --- | --- |
| Overall  (n = 25) | - Patient and family partner: 5 - Clinician: 9 - Manager or Director: 9 - Executive: 2 | Mean: 9.08  Median: 7 | Mean: 47.1  Median: 48.5 | - Woman: 21 - Man: 4 | - Undergraduate, Professional, or Diploma degree: 14 - Graduate degree: 11 | - White: 17 - South Asian: 5 - Black: 1 - Hispanic: 1 - Japanese: 1 |
| Partners (n = 5) | NA | Mean: 6  Median: 5 | Mean: 55.6  Median: 53 | - Woman: 4 - Man: 1 | - Undergraduate, Professional, or Diploma degree: 1 - Graduate degree: 4 | - White: 3 - South Asian: 1 - Black: 0 - Hispanic: 0 - Japanese: 1 |
| Clinicians (n = 9) |  | Mean: 9.8  Median: 7 | Mean: 42.7  Median: 43 | - Woman: 8 - Man: 1 | - Undergraduate, Professional, or Diploma degree: 6 - Graduate degree: 3 | - White: 5 - South Asian: 3 - Black: 1 - Hispanic: 0 - Japanese: |
| Managers and Directors (n = 9) |  | Mean: 9.8  Median: 9 | Mean: 47.25  Median: 50 | - Woman: 8 - Man: 1 | - Undergraduate, Professional, or Diploma degree: 3 - Graduate degree: 6 | - White: 7 - South Asian: 1 - Black: 0 - Hispanic: 1 - Japanese: 0 |
| Executives (n = 2) |  | Mean: 10.5  Median: 10.5 | Mean: 45.5  Median: 45.5 | - Woman: 1 - Man: 1 | - Undergraduate, Professional, or Diploma degree: 1 - Graduate degree: 1 | - White: 2 - South Asian: 0 - Black: 0 - Hispanic: 0 - Japanese: 0 |
| Case 1: Oncology  (n = 11) | - Patient and family partner: 3 - Clinician: 6 - Manager: 1 - Director: 1 | Mean: 9.7  Median: 7 | Mean: 48.7  Median: 51.5 | - Woman: 11 - Man: 0 | - Undergraduate, Professional, or Diploma degree: 8 - Graduate degree: 3 | - White: 6 - South Asian: 4 - Black: 0 - Hispanic: 0 - Japanese: 1 |
| Case 2: Mental Health  (n = 10) | - Patient and family partner: 2 - Clinician: 3 - Manager: 4 - Director: 1 | Mean: 7.7  Median: 6 | Mean: 45.4  Median: 48.5 | - Woman: 7 - Man: 3 | - Undergraduate, Professional, or Diploma degree: 4 - Graduate degree: 6 | - White: 7 - South Asian: 1 - Black: 1 - Hispanic: 1 - Japanese: 0 |

# Healthcare Logics in Organizational Documents

| **Healthcare Logics** | **Strategic Plan** | **MVV Statements** | **Declaration of Respect** | **Patient Declaration of Values** |
| --- | --- | --- | --- | --- |
| **Public Management**: Community engagement, and equity, diversity, and inclusivity. | One call to action, one elaborated vision statement, and one priority that describes the importance of internal and external partnerships, a transformation of health service delivery across all levels, and shaping a more equitable and healthier future. | The mission statement, vision statement and one belief that focuses on collaborative, inclusive, and effective health services aimed at enhancing overall community well-being. | - None | - None |
| **Market**: Sustainability and fiscal performance. | One goal that mentions sustainability of health services. | - None | - None | - None |
| **Medical Professional**: Access, care quality, teamwork, accountability, and mutual trust and respect. | One call to action, two goals, and one priority that emphasize high-quality care, broadening availability, and focusing on delivering outstanding care and experience. | Two values and two beliefs that convey a commitment to superior performance and courage, valuing collective collaboration and fostering a culture of inquisitiveness, innovation, and ongoing development. | Five statements from the declaration of respect that encompass the importance of treating others with the dignity and consideration they desire, actively listening and engaging in dialog to foster trust and mutual comprehension, the value of collaborative teamwork, taking responsibility for one’s actions and their effects on others, and a commitment to learning from experiences for ongoing improvement. | - None |
| **Care Professional / Partnership**: Partnership, engagement, and exceptional care experiences | Two calls to action that advocate for holistic patient care and positive healthcare experiences and two priorities that include excellence in treatment and fostering collaboration with patients to enhance overall health status. | The vision statement, one value, and one belief that emphasize compassionate collaboration and active involvement of patients and their families in care decisions. | - None | Five statements from the patient declaration of values that encompass commitment to providing prompt, high-quality and safe care; informing patients to enable educated decisions; involving patients and significant others in care; building trust through attentive listening and response; and treating patients with respect, compassion and dignity. |

*Due to the volume of excerpts, the content has been summarized and paraphrased in this table.

# Healthcare Logics Across Participant Groups

| **Healthcare Logics** | **Patient and Family Partners** | **Clinicians** | **Managers** | **Directors and Executives** |
| --- | --- | --- | --- | --- |
| **Public Management**: Community engagement, and equity, diversity, and inclusivity. | - Partners indicated a need to understand different community groups' unique needs and experiences. - Partners emphasized the importance of mirroring the community's diversity within the hospital’s PFACs. - Partners suggested a focus on ensuring that all segments of the community, especially underrepresented groups, have a voice in healthcare planning and policy-making. | - Clinicians emphasized the hospital's vision of creating a healthier community. From their perspective, this vision reflected a strategic focus on community well-being. - Clinicians believed in integrating PE in the mission statement as it relates to strengthening the community. According to them, integrating PE is crucial for improving patient satisfaction and enhancing the hospital's ability to serve the community more effectively. - Clinicians believed in the strategic plan's focus on community health. Clinicians perceived this as including patients and their families in discussions and decision-making processes, thereby prioritizing community-centric health solutions. - Clinicians viewed the directive to treat local patients within the hospital, even when facing capacity limits, illustrates a commitment to the public management logic. | - Managers emphasized the importance of connecting with the community to understand and address its diverse healthcare needs. They recognized the necessity of forming partnerships with community organizations and analyzing demographic data to ensure that the hospital's services align with the community's health needs. - Managers acknowledged the need for embracing diversity and inclusivity in healthcare. Managers appreciated the hospital's dedication to anti-racism and equity, which they viewed as integral to improving patient experiences. This commitment reflects an understanding that healthcare delivery must be responsive to the diverse backgrounds and needs of patients and families. - Managers highlighted the hospital's goal of considering the diversity of the patient population. This involved adopting a holistic approach to patient care that takes into account the varied backgrounds and experiences of patients. They acknowledged the importance of partnering with patients and families in a way that respects and responds to the diversity of the communities they serve. | - Directors and executives stressed the importance of understanding the community's health needs, highlighting a focus on population health as a central goal. The desire to integrate community feedback into strategic planning demonstrates an overarching commitment to aligning hospital initiatives with public health priorities. - Directors and executives emphasize incorporating community voices in the decision-making process, ensuring that healthcare services and strategies are reflective of community needs. Efforts to actively partner with various community segments, especially those traditionally underrepresented or in lower socio-economic areas, are aimed at ensuring comprehensive representation and equity. - Directors and executives indicated a strong dedication to becoming an anti-racist organization with measurable goals in equity, diversity, and inclusivity. This commitment extended to PE practices, where diverse perspectives are actively sought to influence hospital operations and patient care strategies. - Directors and executives articulated the integration of equity, diversity and inclusivity considerations into practical aspects of healthcare delivery, such as the design of communication tools like patient room whiteboards. The involvement of patients and families from various races and ethnicities in these design processes exemplifies a practical approach to embedding inclusive practices within hospital operations. - Directors and executives viewed the hospital as a community resource, deeply intertwined with the needs and values of the community it serves. This vision aims to create a health system that speaks the same language as its users, ensuring that community needs and preferences are central to the hospital’s identity and functioning. - Efforts are being made to develop a workforce that mirrors the diversity of the community, enhancing the hospital's ability to understand and cater to varied patient needs. This strategy is seen as crucial for providing safe and inclusive care environments, reinforcing the hospital’s role as a diverse and community-centric organization. |
| **Market**: Sustainability and fiscal performance. | - None | - None | - Managers emphasized the importance of sustainability in their program planning process and reported evaluating new projects and groups with a lens on their sustainability. This indicates a commitment to long-term planning and efficient resource management. | - None |
| **Medical Professional**: Access, care quality, teamwork, accountability, and mutual trust and respect. | - None | - Clinicians were acutely aware of the importance of quality care to the hospital. In particular, clinicians highlighted patient satisfaction as a central goal of their work. - Clinicians acknowledged the goal of providing timely access to health services. However, they also discussed the practical challenges in meeting these goals, such as limitations in access to tests or operational constraints. - The use of communication tools like AIDET and SOAP were highlighted by clinicians as part of their goal-oriented approach. The use of these tools demonstrated a structured and patient-centered method in interactions, aiming to improve clarity and effectiveness in patient care. - Clinicians mentioned the role of ongoing training and education, particularly in light of hospital accreditation. The emphasis on continuous learning and adapting to new practices underscored a commitment to maintaining high care standards. - For clinicians, regular meetings with clients and the arrangement of family meetings when necessary signified an approach deeply embedded in the ethos of medical professionalism. | - Managers prioritized quality indicators as essential tools in program evaluation and improvement. This focus on measurable outcomes such as care planning, patient belongings management, and violence assessment tools reflected a dedication to refining healthcare delivery and ensuring patient safety. - Managers evaluated new projects and initiatives based on their potential to enhance the quality of care. This approach illustrated a deep-rooted commitment to maintaining high standards of healthcare delivery, ensuring that all new initiatives aligned with the hospital’s overarching aim of providing high quality care. - Managers uniquely integrated considerations of quality, access, and sustainability in new initiatives and programs. - Managers described a commitment to improving the health and well-being of patients. - Managers emphasized the right of patients to access healthcare, highlighting the importance of patient empowerment and education in healthcare settings. | - Directors and executives emphasized a strategic commitment to delivering the highest quality of care coupled with exceptional patient experiences. |
| **Care Professional / Partnership**: Partnership, engagement, and exceptional care experiences | - Partners highlighted a strong focus on the patient experience from the moment of entry into the hospital. This approach underscored the emphasis on patient well-being at every step of their journey within the hospital. - Partners described their involvement in organizational activities like accreditation, reflecting a commitment to integrating patient and family perspectives in hospital governance and decision-making processes. - Having staff acknowledge PFACs goals illustrates structured and purposeful PE. - Partners believed that the hospital valued patient and family opinions, and this belief reinforced a sense of partnership and shared decision-making. It indicated a culture where patient and family input are not only sought but also respected. | - Clinicians embraced a person-centered care philosophy. They believed that this philosophy was ingrained in the hospital's culture, ensuring that patients have a say in all aspects of their care. It emphasized the value of patient input in healthcare decision-making and operational activities. - Clinicians acknowledged the importance of engaging families, recognizing that patients' well-being is often closely tied to their familial support system. This approach underlines the commitment to holistic care, considering the social and emotional contexts of patient health. - Clinicians expressed a commitment to compassion in patient care but also revealed concerns about achieving excellence in clinical services. This dichotomy highlighted the ongoing struggle to balance empathetic, person-centric care with clinical standards. - There was a belief among clinicians that PE is a key focus in the hospital. Clinicians see themselves as vital in bridging these values in patient care. - Patient and family engagement was identified as a significant priority during accreditation. - Clinicians mentioned specific practices like providing patient pamphlets and encouraging participation of patients and families in unit huddles. | - Managers focused on considering the whole person, which involved recognizing and addressing the diverse needs of the patients and their families. This approach went beyond treating the illness to understanding the patient's broader life context, including their social, emotional, and cultural dimensions. - Managers recognized the overarching goals of the hospital in terms of quality, access, and sustainability. They aligned PE with these goals, aiming to deliver high-quality healthcare that leads to improved patient experience. - The implementation of terms of reference and a values framework, developed in collaboration with patient and family partners, guides the evaluation of PE initiatives. This structured approach ensures that PE is respectful, meaningful, and valuable. - Managers expressed a clear goal to partner with patients and consider the diversity of the population. This reflects an inclusive approach to healthcare, where patient and family input is integral, and care is tailored to the unique needs of each individual, respecting their diverse backgrounds and experiences. | - Directors and executives emphasized the importance of exceptional patient experiences as a core element of their strategic plan, aiming to integrate PE in the design and implementation of hospital processes. - The focus extends beyond strategic objectives to practical applications, such as incorporating patient-centered care in daily practices. Examples include engaging patients and families in transfer of accountability during shift changes, indicating an operationalization of patient-centric values in clinical routines. - The goal of making PE meaningful was stressed by directors and executives. It was not just about involving patients and families but doing so in a way that is genuinely valuable and impactful for both the patients and the hospital. - The selection of accreditation standards with a strong focus on person-centered care demonstrated a formal commitment to PE. - There was a deliberate effort to quantify patient experience through metrics like patient recommendation rates. Setting formal goals, such as achieving an 80% recommendation rate, illustrates a structured approach to monitoring and enhancing patient experience. |

Goals often intertwined and were connected to practices, processes and activities. For the purposes of this analysis, goals were separated from practices, processes and activities to deeply analyze how goals are understood and described by participants.
